# Ophthalmology and Surgery

**Published:** 1876-01

**Authors:** 


### III. OPHTHALMOLOGY AND SURGERY.

1. *Two Cases of Aneurisma Arterioso-Venosum in the Orbit.* RIVINGTON. (*La France Médicale*, No. 71.)

In a recent meeting of the Med. and Chir. Society of London, Walter Rivington reported the following case: In July, 1873, a young man was admitted to the London Hospital with a fracture of the skull. Six weeks later he began to hear a blowing noise in his head; the eyeball became slightly prominent, and then a pulsating tumor appeared between the globe and orbital border, chemosis, œdema of the lids and paralysis of the muscles of the eyes ensued. After an ineffectual trial of digital compression of the carotid, and after an unsuccessful injection of perchloride of iron, the carotid was ligated. The patient recovered, but the sight of the eye was greatly damaged by a superficial ulceration of the cornea that came on a few days after the ligation. The doctor diagnosticated an aneurisma arterioso-venosum, caused by a communication of the internal carotid with the cavernous sinus in consequence of the fracture of the skull.

Soon after this, P. Lansdown published (*British Med. Journal*, June 5,) a similar case. A laborer had the inner half of his left upper lid cut across, by a piece of an exploded seltzer bottle. L., who at once saw the patient, found a bleeding artery and the lid distended by copious effusion of blood into the cellular tissue; the hæmorrhage was arrested by uniting the borders of the wound with a suture, and the patient appeared to be doing well. After six weeks, however, the eye had become somewhat protruding, the lids were swollen and the veins of the conjunctiva appeared tortuous and dilated. And several weeks later a small pulsating tumor was discovered at the inner canthus, below the cicatrix of the upper lid. Over the whole globe one could hear a bruit which disappeared on compression of the carotid. The sight was intact. An instrument was applied by which the aneurism was pressed against the inner wall of the orbit; but this treatment had to be abandoned on account of the pain caused by the compression. From the fact that the retinal veins were dilated like the conjunctival vessels, several surgeons were led to diagnosticate an aneurism in the depth of the orbit, and advised the ligation of the carotid. But L. preferred the immediate ligation of the aneurismal artery, considering this operation more satisfactory and less dangerous than the ligature of the carotid, even if he had to enucleate the globe to accomplish his purpose. An incision made into the old scar, exposed a small, round, pulsating tumor of the size of a large pea. A large tortuous vein emerging from the depth of the orbit, clung tightly to the anterior surface of the tumor. A communication between the nasal artery and vein had given rise to the deformity of the eye. The artery found, though with considerable difficulty, and separated, was ligated at both sides of the aneurism. On the fourth day the sac was closed, and at the end of one week the globe had recovered its normal position. And now, one year after the operation, the conjunctival as well as the retinal veins show a normal calibre and no difference between the two eyes can be noticed.

2. *Miners' Nystagmus.* TAYLOR. (*Am. Jour. Med. Sci.*, Oct., 1875 )

Dr. Chas. B. Taylor describes (*Lancet*, June 12, 1875,) under this name a peculiar malady of which the mines in the neighborhood of Nottingham have furnished him several examples. It is characterized by peculiar oscillating motions of the eyeballs, and has been observed only in adults, and, independent of other ocular defects, in men employed in coal pits. The oscillating movements are caused by alternating contractions of the recti or oblique muscles, and are horizontal or rotatory. This affection appears to be analogous to writers' cramp or similar affections, and is caused by an overtasking of the muscles of the eye. By a great and sustained effort of the patient to see in an imperfect light, they are overburdened, in the course of time give way, and at last, whenever called upon, become, as it were, agitated and fluttered, escape from the control of the will and perform irregular motions. As a rule, change of occupation and working in a good light are all that is necessary to effect a cure.

3. *Extirpation of the Larynx.* (*Am. Jour. Med. Sci.*, Oct., 1875.)

The first extirpation of the larynx in man, was performed by Billroth (Vienna), in December, 1873; he was followed by Heine (Prague) and Moritz Schmidt (Frankfort). And on July 21st last, Prof. Von Langenbeck (Berlin) excised the larynx with the hyoid bone and a portion of the tongue, pharynx and œsophagus for cancerous infiltration of the parts mentioned. The patient, aged fifty seven years, was kept completely unconscious for two hours, the chloroform being administered through a canula introduced into the trachea below the larynx; above the canula the trachea was plugged with an air-bag, so as to prevent any downflow of blood into the bronchi. Forty-one arteries were ligated, including both external carotids. After the operation the immense wound was simply closed by compresses soaked in a one-third per cent. solution of salicylic acid, and no attempt was made to bring the skin together with sutures. Up to July 28, the patient did well and had no fever.

4. *Treatment of Diphtheria.* THEO. SCHUELER. (*Berl. Klin. Wochenschr.*, 1875, No. 40.)

The writer treated 79 cases, and in order to ascertain the effect of different remedies, he successfully tried chlorate of potassium, carbolic acid and salicylic acid. He lost six patients of 41 treated with chlor. potass., one of 23 treated with carbolic acid, and seven of 15 treated with salicylic acid. These results do not speak in favor of the salicylic acid.

5. *How to Prevent Chloroform Asphyxia.* Dr. J. FLEIBERG. (*Berl. Klin. Wochenschr.*, 1875, Sept.)

His plan is to dislocate the inferior maxillary bone forward whenever any asphyxial symptoms make their appearance. The best way of producing this dislocation is to stand behind the reclining patient, put both thumbs behind the symphysis and the index fingers on the posterior edges of the rami of the bone, then grasp the maxilla firmly and drag it directly forward. If the patient be under the influence of the anæsthetic—and then only it is necessary to resort to this procedure—the condyle will move forward with a perceptible motion and the entire bone is displaced. As soon as this is done the patient takes a long breath and respiration proceeds without any further difficulty as long as the parts remain in the same position. The author has employed this procedure in more than a thousand instances and has never failed to achieve the desired purpose, namely, the use of chloroform without any unpleasant complications. He believes that the root of the tongue and the epiglottis are dragged forward and thereby the occlusion of the larynx and the consequent asphyxia which is due to the weight of the tongue, are obviated.

The same method has been in constant use by Esmarch since 1866; and Langenbeck employed it constantly during the late Franco-German war, and never lost a case from the effects of chloroform.

6. *A New Method of Controlling the Velum Palati in Rhinoscopic Exploration.* PH. S. WALES. (*N. Y. Med. Record*, 1875, No. 48.)

After giving a brief résumé of instrumental and other means which have been devised (by Czermak, Turck, Voltolini and others,) to control the movements of the palate, the writer continues:

"My principle consists essentially in overcoming the contraction of the palatal muscles by elastic force, and the means of fully carrying it out will be found in an india-rubber cord. The simplest method of putting it in position after having selected one of such a diameter—two millimetres will do—as will readily pass through the inferior meatus into the pharynx without any instrumental assistance, is the following:

"One end is introduced into each nostril, until they both reach the lower portions of the pharynx. At this moment the patient is directed to cough, if the presence of the thread has not already excited this movement; the force of expiration will pretty surely project them into the mouth, when they may be apprehended with the fingers and drawn externally until the middle portion of the cord, which is external, is arrested against the nasal septum. Gentle traction is continued until the soft palate is well drawn forward, when the threads are passed up over the ears, and downwards beneath the chin and there tied, or they may be held by the patient himself. At any moment after the ends of the elastic are secured at the point indicated, the tension of the cord and correlative palatal pressure may be increased by seizing the threads as they pass out of the mouth and gently drawing them forward, until the palatal contraction is entirely overcome, and the area of the pharyngo-buccal space ample enough to receive the largest mirror. It will sometimes be observed that where there is very much irritability, the velum palati momentarily contracts, especially at the time when the mirror is introduced, but soon yields to the elastic force of the thread. Should any impediment whatever exist in the nostrils, that the cord cannot be passed by itself, the little instrument I have described below works admirably as a cord carrier. Of course, an expert hand may make use of any instrument that may chance around a catheter, slips of whalebone, or wood.

"The device mentioned above is simple, cheap, and easily managed. It is a thin lamina of soft metal, six inches long and less than an eighth wide, mounted at each extremity with a small ring of an amplitude a little greater than the elastic cord, which having been passed through them, is tipped with small, smooth, oblong fragments of lead. When the instrument is to be used, the cord is drawn through the rings until one of its tips comes against the corresponding ring; slight tension of the elastic will retain the two in contact while the point thus formed is being conducted along the inferior meatus. When the metallic point reaches the posterior wall of the pharynx, the elastic projecting externally is pulled through the exterior ring, and made quite slack so that the instrument may be withdrawn from the nares, leaving the cord in position; a similar proceeding is then practiced upon the other nostril.

"The metallic points of the cord, now in the pharynx, may be easily thrown forward by coughing or hawking, and seized with the fingers and drawn externally. The metallic lamina, on account of its flexibility, will thus be bent as a bow over the bridge of the nose, and out of the way.

"The discovery of means of amplification of the pharyngo-buccal aperture has occupied my attention for several years. I have had only moderate success until the discovery of the devices I now bring to the notice of the profession.

"Its chief merits are the simplicity of the apparatus, and the facility with which any professional person may employ it in exploring the posterior nares and the pharyngeal cavity. The cord itself may well take the place of Belloc's sound in any case wherein it may be necessary to conduct a thread through the nostrils, as in plugging the nares for epistaxis. Here, however, I employ a little bulb of rubber at the end of a hollow rubber cord, and after the bulb is placed in position through the fauces, it may be inflated by blowing air through its open extremity which projects exteriorly from the nose."

7. *Osteitis of Pearl-Turners.* C. GUSSENBAUER. (*Langenbeck's Archiv.; Allg. Med. Centralz.*, 1875, No. 85.)

This disease, first described by English, was observed in Billroth's clinic, by C. G. on six young men who had been working on mother-of-pearl. They were all six yet in the period of development; and those who continued their occupation were often subject to a recurrence of the disease. The seats of the malady were the lower jaw, scapula, humerus, ulna, radius, femur, fibula, os calsis, cuboideum, and metatarsal bones. The osteitis begins when the youth has been working in the factory for several months. First a slight fever occurs, and a pain which is located precisely in the very seat of the inflammation. After a few days a periosteal swelling appears, which, at first soft, elastic, sometimes fluctuating, and very tender, may afterwards assume the hardness of bone. The tissues covering the affected bone become swollen a few days after the appearance of the periosteal tumor. It is a significant characteristic of these tumors that they are seated in the long bones, always near one or the other end of the diaphysis and never occupy the middle or the epiphysis. The tumefaction gradually increases in size, yet it never terminates in suppuration, but is reabsorbed under the local use of mercurial ointment, warm poultices, and the internal administration of iodide of potassium. The writer ventures an explanation of this peculiar disease and attributes it to an embolic process: the workmen are employed in small compartments, the air of which is filled with the dust of mother-of-pearl; this dust (containing 93.5 per cent. of carbonate of lime and 6.5 per cent. of organic matter,) is inhaled into the lungs, from there it may get into the circulation, and finally happen to stop in the small nutritive arteries of the bones, giving rise to thrombosis infarctus and consecutive osteitis and periostitis. As to the inhalation of the dust, he shut up some dogs for a few days in a box, the air of which was kept loaded with the dust of mother-of-pearl. When these dogs were killed after some time, tubercles (the largest of the size of a wheat grain) of such dust were found in the parenchyma of the lungs.

8. *Results of Antiseptic Treatment.* A. BARDELEBEN. (*Centralbl. f. Chir.*, 1875, No. 47.)

In a paper read before the Berlin Med. Society, Prof. Bardeleben gave an account of 387 cases treated antiseptically during the past three years. Among these cases there were 134 large abscesses, 56 extensive phlegmonous infiltrations, 76 major amputations, 21 major excisions of joints, 23 extirpations of large tumors, 77 complicated fractures. The result of the treatment was that none of the patients died of pyæmia or septicæmia, and that erysipelas though still appearing occasionally, seemed to be of a milder character.

9. *A Rare Tumor of the Mesentery.* A. WEICHSELBAUM. (*Virchow's Arch.* LXIV; *Centralbl. d. Chir.*, 1875, No. 43.)

Between the layers of the very fatty mesentery of a man, 80 years of age, a tumor of the size of the palm of a hand was found. A fluid which proved to be chyle, by chemical and microscopic examination, ran from the cut surface as though it were oozing from a sponge. The tumor showed the structure of a cavernous angioma, but its containing chyle matter proved that its cavernæ communicated with the lymphatic vessels of the mesentery. Originally the neoplasm was most likely a lipoma, in which the cavernous lymphatic spaces were gradually developed; its periphery, at least, was constituted of adipose tissue.

10. *Encephaloid Cancer.* C. V. MOLTRAM, M.D. (*Transac. Kansas Med. Soc.*, 1875.)

In Aug., 1872, a girl of 14, "bumped" her nose against a table. it soon recovered, and was "bumped" against a baby's head in Sept.,1872; a slight swelling followed and remained. In Dec. it grew worse, and by New Years the nose, from the end of the nasal bone to the os frontis, had greatly enlarged. The superior and middle turbinated bones were involved, filling the nostrils. The disease progressed most between the eyes, which were forced forward and outward. By February, 1874, the space between the inner canthi was 3½ inches. By June, the tumor extended from the tip of the nose upward 9 inches, laterally 9 inches, and antero-posteriorly 3 inches. The eyes were forced out of their sockets upon the temples, yet they retained the sight, and the lachrymal ducts were pervious. A month or two previously an abscess had formed in the left mammary gland and opened spontaneously without pain. Her appetite and digestion were good to within a few days of her death, when diarrhœa commenced. She slept well. The parotid, submaxillary and lymphatic glands of the neck became enlarged late in the case, and a tumor appeared near the sternum between the first and second ribs, of the size of a turkey's egg, and she had a slight cough. She died October 1st. The tumor was found made of "apparently elastic fibrous tissue and yellow-white brain-like substance," which fluctuated easily. Weight 5 lbs. The tumor near the sternum communicated with larger tumor within thorax. The right lung was *solidified* with cancerous deposit; the left lung was more than half full of the same deposit. The mesentery was studded with hard deposits. Microscopic examination revealed cancer cells in all the morbid parts.

